# Drug‐Induced Differential Gene Expression Analysis on Nanoliter Droplet Microarrays: Enabling Tool for Functional Precision Oncology

**DOI:** 10.1002/adhm.202401820

**Published:** 2024-10-23

**Authors:** Razan El Khaled EL Faraj, Shraddha Chakraborty, Meijun Zhou, Morgan Sobol, David Thiele, Lilly M Shatford‐Adams, Maximiano Correa Cassal, Anne‐Kristin Kaster, Sascha Dietrich, Pavel A. Levkin, Anna A. Popova

**Affiliations:** ^1^ Institute of Biological and Chemical Systems‐Functional Molecular Systems Karlsruhe Institute of Technology Hermann‐von‐Helmholtz‐Platz 1 76344 Eggenstein‐Leopoldshafen Germany; ^2^ Department of Biochemical Engineering and Biotechnology Indian Institute of Technology Delhi New Delhi 110016 India; ^3^ Institute for Biological Interfaces 5 Karlsruhe Institute of Technology Hermann‐von‐Helmholtz‐Platz 1 76344 Eggenstein‐Leopoldshafen Germany; ^4^ Department of Biology Doane University Crete NE 68333 USA; ^5^ Department for Hematology Immunology and Clinical Oncology Heinrich‐Heine‐University Düsseldorf 40225 Düsseldorf Germany

**Keywords:** chronic lymphocytic leukemia, differential gene expression analysis, droplet microarray, drug screening, functional precision oncology, qPCR

## Abstract

Drug‐induced differential gene expression analysis (DGEA) is essential for uncovering the molecular basis of cell phenotypic changes and understanding individual tumor responses to anticancer drugs. Performing high throughput DGEA is challenging due to the high cost and labor‐intensive multi‐step sample preparation protocols. In particular, performing drug‐induced DGEA on cancer cells derived from patient biopsies is even more challenging due to the scarcity of available cells. A novel, miniaturized, nanoliter‐scale method for drug‐induced DGEA is introduced, enabling high‐throughput and parallel analysis of patient‐derived cell drug responses, overcoming the limitations and laborious nature of traditional protocols. The method is based on the Droplet Microarray (DMA), a microscope glass slide with hydrophilic spots on a superhydrophobic background, facilitating droplet formation for cell testing. DMA allows microscopy‐based phenotypic analysis, cDNA extraction, and DGEA. The procedure includes cell lysis for mRNA isolation and cDNA conversion followed by droplet pooling for qPCR analysis. In this study, the drug‐induced DGEA protocol on the DMA platform is demonstrated using patient‐derived chronic lymphocytic leukemia (CLL) cells. This methodology is critical for DGEA with limited cell numbers and promise for applications in functional precision oncology. This method enables molecular profiling of patient‐derived samples after drug treatment, crucial for understanding individual tumor responses to anticancer drugs.

## Graphical abstract

A novel miniaturized method for nanoliter‐scale drug‐induced differential gene expression analysis is presented. Using the droplet microarray platform, this approach enables high‐throughput analysis of patient‐derived cells, overcoming the challenges associated with limited samples. This technique provides critical insights into individual tumor responses, advancing the field of functional precision oncology.

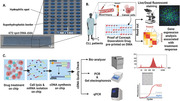

## 1 Introduction

In the dynamic field of cancer research and treatment, differential gene expression analysis (DGEA) plays a crucial role. The study of gene expression patterns through transcriptomic analysis is used to decipher the molecular signatures that characterize different types of cancer or the mechanism of drug response and resistance of individual tumor types.^[^ ^1^, ^2^, ^3^ ^]^ The transcriptional responses of cells show variations across different tissues, different physiological conditions, and in response to environmental cues.^[^ ^4^, ^5^ ^]^ Initially, transcriptome analysis aimed to identify differentially expressed genes, and various methods were developed to analyze the transcriptome to identify genes with significant changes in their expression. Early techniques such as expressed sequence tag (EST), serial analysis of gene expression (SAGE), and hybridization‐based gene microarray or chip technology played a key role in providing rapid insights into gene expression in different biological contexts.^[^ ^2^ ^]^ Subsequently, microarray technologies played a critical role in unravelling gene expression patterns, providing valuable insights into the molecular complexities of cancer.^[^ ^6^, ^7^ ^]^ Finally, the advent of next‐generation sequencing (NGS), particularly RNA sequencing (RNA‐seq), revolutionized transcriptome analysis by providing high‐throughput and accurate quantification of gene expression.^[^ ^5^, ^8^ ^]^ Understanding gene function in a physiological context begins with the study of gene expression, where reverse transcription‐quantitative real‐time PCR (RT‐qPCR) assays offer reproducible, quantitative, and rapid analysis. RT‐qPCR is widely used for quantitative gene expression analysis in various fields, such as molecular biology, medicine and diagnostics, offering the ability to compare mRNA expression levels in different biological samples and to confirm findings from other techniques, such as microarrays or next‐generation sequencing due to its high sensitivity, specificity, and reproducibility.^[^ ^2^, ^9^ ^]^

Modern methods for DGEA are evolving to require less cell input for analysis, even down to the single cell level, while focusing on miniaturization and parallelization of sample preparation protocols.^[^ ^7^, ^10^, ^11^ ^]^ However, single‐cell RNA sequencing presents significant challenges, with accurate measurements dependent on enzymatic efficiency and an amplification step that is prone to error.^[^ ^12^ ^]^ To address the inherent challenges of single‐cell analysis, new technologies have emerged that overcome the limitations of traditional single‐cell sequencing methods and provide comprehensive workflows for capturing and analyzing gene expression profiles at the single‐cell level. By integrating advanced microfluidic and molecular biology techniques, it is now possible to acquire high‐quality data from single cells with improved sensitivity and accuracy. These advances are not only enriching our understanding of cellular heterogeneity and gene regulation but are also driving significant breakthroughs in disciplines, such as cancer biology, developmental biology, and immunology.^[^ ^7^, ^13^, ^14^ ^]^ For example, Streets et al. developed a method for sequencing single cell messenger RNA (ScmRNA). They implemented a microfluidic valve‐based technique in which their platform captures single cells, lyses them and performs reverse transcription of mRNA into cDNA. The resulting single‐stranded cDNA (sscDNA) is then transferred to conventional PCR tubes for amplification and purification, facilitating subsequent sequencing to analyze the gene expression profile.^[^ ^7^ ^]^ Using droplet microfluidics, Klein et al. developed inDrop (indexing droplets) RNA sequencing, a technique capable of indexing thousands of individual cells for RNA sequencing. The inDrop platform encapsulates cells in droplets containing lysis buffer, reverse transcription (RT) reagents, and barcoded oligonucleotide primers. Within each droplet, mRNA released from the lysed cell is barcoded during the synthesis of complementary DNA (cDNA). After barcoding, material from all cells is combined by breaking the droplets, and the cDNA library is sequenced using established methods (CEL‐Seq). To ensure that each droplet carries primers encoding a different barcode, Klein et al. synthesized a library of barcoded hydrogel microspheres (BHMs) coencapsulated with cells.^[^ ^10^ ^]^ Microwell‐based platforms, as demonstrated by Bose et al. are being used in various fields including cell culture, image analysis, and single cell RNA sequencing. In this context, the microfluidic device traps single‐cell lysates in microwells, where mRNA molecules released from the lysed cells hybridize to oligo(dT) primers on the glass surface, forming single‐cell mRNA prints. After sealing, on‐chip reverse transcription converts the mRNA prints to cDNA, which is then stained with SYTOX Orange for fluorescence imaging, allowing visualization of gene expression patterns at the single cell level.^[^ ^15^ ^]^ Yuan et al. used subnanoliter wells to capture cells, barcode them using poly(dT) mRNA capture beads, and perform subsequent on‐ and off‐chip procedures, including reverse transcription, cDNA amplification, library preparation, and paired‐end sequencing.^[^ ^6^ ^]^ All of these methods play a critical role in the analysis of differential gene expression at the single cell level within a given cellular state. Cells or pre‐existing mRNA are introduced into these platforms from external sources, such as cells extracted from different tissues or subjected to drug treatments using different platforms, such as multiwell plates. None of these platforms integrate both drug treatment and sample preparation.

Open droplet microarrays (DMAs) offer significant advantages over traditional platforms, such as multiwell plates and microfluidic systems, making them ideal for high‐throughput and flexible experimental applications. Unlike traditional platforms, DMAs can efficiently manage thousands of nanoliter droplets on an open array, enabling large‐scale screening for drug discovery,^[^ ^16^, ^17^ ^]^ personalized medicine,^[^ ^18^ ^]^ cell culture,^[^ ^19^ ^]^ nucleic acid screening,^[^ ^20^ ^]^ and combinatorial chemistry.^[^ ^21^, ^22^, ^23^, ^24^, ^25^ ^]^ This setup supports massively parallel experiments without the need for complicated designs or multiple channels, which are often complex and costly to implement in microfluidic systems. DMA can be easily adapted to different experimental needs without requiring extensive set‐up procedures, allowing easy customization of droplet volume and content.^[^ ^26^ ^]^ This flexibility differs significantly from microfluidic systems, which typically require precise control mechanisms, specialized chips and complex designs that can be expensive and difficult to modify. They also overcome challenges, such as clogging and cross‐contamination commonly associated with microfluidic channels, making them a more accessible option, particularly for laboratories with limited resources.^[^ ^27^ ^]^ The platform's versatility extends to a wide range of assays, from biochemical reactions to cell culture, without the need for significant system redesign, making DMAs as cost effective as they eliminate the need for custom chip fabrication for different purposes and experimental needs.^[^ ^28^ ^]^

DMAs integrate seamlessly with standard laboratory equipment, such as microscopes, colorimetric scanners, and automated liquid handlers, improving ease of use and operational efficiency. The integration of AI, machine learning and automation with DMAs further enhances their ability to generate rich experimental data.^[^ ^29^ ^]^ Overall, the open DMA platform provides a more adaptable, efficient, and cost‐effective solution compared to traditional well plates and microfluidic systems, offering significant benefits to researchers seeking high‐throughput capabilities and experimental flexibility.^[^ ^21^, ^22^, ^26^, ^27^ ^]^

Drug‐induced DGEA, especially with the possibility of parallel phenotypic analysis using microscopy, can be an indispensable tool for uncovering the molecular basis of phenotypic changes in cells upon drug treatment and ultimately for understanding the mechanisms of drug response. Postdrug treatment transcriptomics using qPCR and mRNAseq has been applied in multiwell plates,^[^ ^30^, ^31^ ^]^ but it is challenging due to the high cost and labor‐intensive multistep sample preparation protocols. Performing drug‐induced DGEA on cancer cells derived from patient biopsies is especially important, since the insights obtained from molecular profiling of unique patient‐derived samples upon drug treatment in vitro, are indispensable for understanding the individual tumor response to anticancer drugs. However, the use of primary cells poses increased challenges due to the scarcity of available cells, requiring miniaturized and low‐input protocols.

In current study, we demonstrate a methodology for performing drug‐induced DGEA using the DMA platform. The DMA platform consists of an array of nanoliter droplets confined on hydrophilic spots isolated by wall‐less superhydrophobic boundaries. We have previously successfully used the DMA platform for cell‐based screening applications in both 2D and 3D environments.^[^ ^16^, ^18^, ^19^, ^20^, ^31^, ^32^, ^33^, ^34^, ^35^ ^]^Although the DMA was demonstrated as a platform for testing a small number of cells with compounds followed by phenotypic analysis through microscopy, it has not been previously utilized for drug‐induced DGEA.

We have previously demonstrated a protocol for mRNA isolation from cells on the DMA chip using oligo d(T) magnetic beads followed by conversion to cDNA using as low as a single cell.^[^ ^20^ ^]^ In this study, we have refined the protocol by eliminating the need for oligo d(T) magnetic beads, thereby further simplifying the process and for the first time have demonstrated the ability to detect changes in gene expression following drug treatment on a chip, demonstrating the application of the protocol on patient‐derived chronic lymphocytic leukemia (CLL) cells. CLL is a type of cancer that affects the blood and bone marrow and is characterized by an accumulation of abnormal lymphocytes in the body. The disease usually progresses slowly and may not require immediate treatment in its early stages. However, as the disease progresses, treatment options become more critical.^[^ ^36^, ^37^ ^]^

However, the treatment options available for CLL, including chemotherapy, targeted therapy, and immunotherapy, are very limited and have various side effects. For example, common treatments include drugs, such as fludarabine, cyclophosphamide, and rituximab, which can be effective but can also lead to side effects and resistance. Targeted therapies, such as ibrutinib and Venetoclax offer more specific approaches by targeting specific molecules involved in cancer cell growth, with ibrutinib inhibiting Bruton's Tyrosine Kinase (BTK), a key enzyme in B‐cell receptor signalling, and Venetoclax blocking BCL‐2, a protein that prevents apoptosis, thereby disrupting crucial survival pathways in cancer cells. Despite these advances, resistance to treatment is a major challenge, with some patients developing resistance to these therapies over time. This resistance can occur through various mechanisms, such as mutations in the target proteins or activation of alternative signaling pathways. Clinical trials are underway to explore new treatment strategies and combinations to overcome resistance and improve patient outcomes.^[^ ^38^, ^39^, ^40^ ^]^

According to ClinicalTrials.gov, several trials are investigating novel approaches to CLL treatment. For example, in the study NCT04447768, the efficacy of a combination of venetoclax and obinutuzumab is being evaluated in patients with previously untreated CLL, and in the study NCT04653536, combinations of targeted drugs (venetoclax, ibrutinib) with anti‐CD20 antibodies (rituximab, obinutuzumab) are being investigated, which may induce extremely long‐lasting remissions. These trials aim to address the limitations of current therapies and improve the long‐term management of the disease.^[^ ^41^, ^42^ ^]^ By miniaturizing the entire workflow from cell culture to cDNA synthesis in nanoliter volumes, reagent and cell consumption were reduced by a factor of 300 and 100, respectively, compared to a 384‐well plate. The methodology established here serves as a critical foundation for performing DGEA on limited numbers of cells, offering potential applications in functional precision oncology.

## 2 Results and Discussions

### 2.1 Concept and Experimental Workflow of Drug‐Induced DGEA on DMA Chip

**Figure 1:**
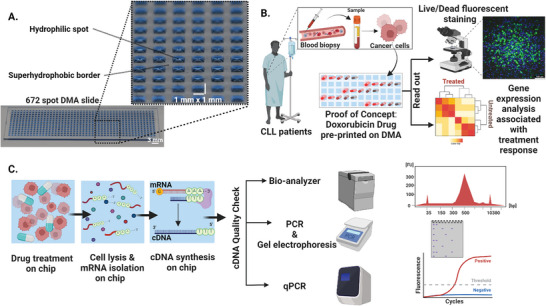
Concept and experimental workflow of drug‐induced DGEA on DMA chip. A) The DMA platform with 672 hydrophilic spots separated by a superhydrophobic background, each spot measuring 1 mm × 1 mm. B) Blood biopsy‐derived CLL cells are applied to individual spots on a preprinted DMA slide. The slide features a proof‐of‐concept drug, Doxorubicin, at five different concentrations, demonstrating the application of a drug library for testing. Following a 24 h incubation period, live/dead fluorescent staining (utilizing Calcein, Hoechst, and propidium iodide) to estimate cell viability and DGEA can be performed to analyze the drug response. C) The sample preparation protocol for DGEA includes the lysis of cells treated with drugs with a lysis buffer, causing the release of mRNA from the cells into the droplets, which then is converted into complementary DNA (cDNA) via reverse transcription. The resulting cDNA is collected from the DMA chip and subjected to quality checks, including capillary gel electrophoresis (Bio‐analyzer), polymerase chain reaction (PCR), and quantitative PCR (qPCR).

In this study, we introduce a concept and methodology for performing drug‐induced DGEA as a read‐out method in addition to microscopy for in‐depth characterization of gene expression changes in cells upon drug treatment, in nanoliter format on the droplet microarray (DMA) platform. **Figure** **1** shows the concept and objectives of this study. The workflow was optimized on DMA, which contains an array of 672 square 1 mm^2^ hydrophilic spots separated by hydrophobic borders. This array allows for the formation of an array of nanoliter droplets, working volume from 150 to 200 nL, on a planar surface, serving as nanowells for cell culture and drug screening (Figure 1A). Due to the dramatic miniaturization of the culturing reservoirs, the DMA platform is advantageous for low cell number experiments, which is essential, for example, in the case of patient‐derived cancer cells. Our study ensures robust and reproducible results through rigorous replication. During protocol optimization, each drug concentration was tested across multiple droplets, with at least three technical replicates conducted. This comprehensive approach resulted in consistently low variability, with gene expression variation remaining below 5%. These outcomes demonstrate the reliability and consistency of our platform, even when using reduced cell input. In this study, as a model cell type we chose patient‐derived CLL. CLL was chosen due to several advantages, notably the ease of obtaining these cells in large quantities compared to other patient‐derived hematological malignancies, such as (MDS) and acute myeloid leukemia (AML).^[^ ^43^ ^]^ Additionally, the abundance and the quantity of available CLL cells allows for more effective protocol optimization and refinement. This is particularly important to improve sensitivity and reliability, especially when optimizing experimental conditions that require multiple technical replicates, such as lysis conditions, procedures, and varying cell numbers for sample preparation. Having a large number of CLL cells allows us to rigorously test and fine‐tune the protocol, ensuring both sensitivity and reproducibility. This extensive testing capability makes CLL an ideal model for refining our methods, ultimately advancing the platform's application in high‐throughput, low‐volume conditions, even when working with limited cell inputs. As shown in Figure 1B, we selected doxorubicin as a proof of concept to test our implemented protocol on the DMA slide to validate that our platform can successfully capture and analyze gene expression changes in response to drug treatment, even within such small volumes. Demonstrating the protocol with a single drug will pave the way for testing additional clinically relevant drugs, such as the BCL‐2 inhibitor venetoclax, the anti‐CD20 monoclonal antibody obinutuzumab, and others.

The drug was printed on the DMA in five different concentrations. Patient‐derived cells were exposed to these varying concentrations of doxorubicin, with multiple experimental repeats conducted to validate the protocol's effectiveness and reliability. Both image‐based analysis, such as live/dead fluorescence staining to determine cell viability, and DGEA can be performed as a read‐out for drug effect (Figure 1B). The protocol for DGEA involves lysis of cells, isolation of mRNA and conversion of mRNA to cDNA, all in nanoliter droplets on the DMA chip (Figure 1C). The cDNA generated from each nanoliter droplet is carefully collected and transferred individually into separate Eppendorf tubes to ensure that the material from each droplet is maintained. With this, it allows the precise amplification of sscDNA generated from individual droplets into dscDNA. The cDNA generated from each nanoliter droplet on the DMA platform was characterized by a capillary gel electrophoresis (Bio‐analyzer), polymerase chain reaction (PCR), and quantitative PCR (qPCR).

### 2.2 Validation of the Protocol for DGEA on DMA Chip

**Figure 2:**
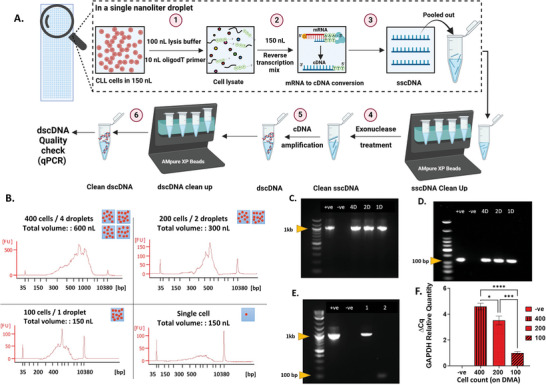
Validation of the protocol for DGEA on DMA chip. A) Schematic representation of the workflow of protocol for cDNA synthesis in individual droplets on DMA slide. B) Qualitative assessment of the cDNA prepared on the DMA chip from cells using the Agilent 2100 Bioanalyzer. This analysis encompasses samples collected from 4, 2, and 1 droplet, containing in total 400, 200, and 100 cells, respectively, as well as a single cell per droplet. Results from a total of three experiments are shown. C–E) Gel electrophoresis of amplified β‐actin (B, product size = 1045 bp) and GAPDH (C, product size = 112 bp) genes from cDNA obtained from 4 (4D), 2 (2D), and 1 (1D) droplet. D) Gel electrophoresis of amplified β‐actin (lane 1, product size = 1045 bp) and GAPDH (lane 2, product size = 112 bp) genes from cDNA obtained from a single‐cell from DMA chip. The “+ve” lane contains positive control, which is cDNA synthesized from SU‐DHL‐4 cells using the standard protocol, whereas the “−ve” lane contains negative control with no template. E) Graph illustrating GAPDH relative gene expression in samples prepared from 4 droplets, 2 and 1 droplets. The error bars represent the mean ± SEM from 3 technical repeats. Statistical significance was determined using ordinary one‐way ANOVA, revealing highly significant differences ****P < 0.0001, ***P < 0.001, *P < 0.01 between consecutive groups, “NS” indicates nonsignificant statistical differences between the analyzed groups.

As a first step, we optimized the protocol for cell lysis and conversion of mRNA into cDNA using the suspension SU‐DHL‐4 cell line. SU‐DHL‐4 is a B‐cell lymphoma cell line, representing one of the most diverse malignancies arising from B‐lymphocytes. Previously, we published a protocol using oligo d(T) magnetic beads for mRNA extraction and purification on DMA chip using adherent cell lines.^[^ ^20^ ^]^ In comparison with the previously published protocol, we removed the step of mRNA purification on DMA chip using oligo d(T) beads, and changed the RNA later lysis (RLT) buffer to Proteinase K enzyme. The current protocol is modified and validated to be suitable for CLL cells, which are suspension cells. The detailed sample preparation protocol is shown in **Figure** **2**. It includes the following steps: 1) lysis of cells using proteinase K and dispensing of oligo‐dT primers, 2) dispensing of RT (Reverse Transcription) mix followed by incubation of the DMA chip at 42 °C for conversion of mRNA to cDNA, 3) collecting the resulting sscDNA (single‐stranded cDNA) synthesized in a total volume of 260 nL from the DMA into a PCR tube, 4) exonuclease treatment, 5) amplification of sscDNA into dscDNA (double‐stranded cDNA), 6) purification of the final DNA product (dscDNA) using AMPure XP beads. As indicated, steps 1 and 2 are performed on the DMA chip, while steps 4 to 6 are performed in a tube using standard protocols. In this study, we manually transferred samples from the DMA chip to the tubes (step 3) using manual pipetting. This is done by adding 10 µL of NFW to each droplet and by pipetting, the content is then collected into PCR tubes. Another method: in one of our recent studies, we demonstrated automated collection and transfer of sscDNA synthesized on the DMA chip using the in‐house developed automated nanoliter droplet selection and collection device ANDeS.^[^ ^29^ ^]^ Automated collection of the droplets opens the possibility of performing high‐throughput workflows on the DMA chip utilizing drug‐induced DGEA as a read‐out.

In order to validate the cell lysis protocol on a DMA platform for efficient mRNA extraction and subsequent conversion to cDNA, a series of experiments were performed comparing different lysis conditions (Figure S1, Supporting Information). The aim was to identify the most effective combination of Proteinase K treatment time, heat inactivation time, both with and without RNAse inhibitor, across different cell numbers. Tube control was performed under standardized conditions (10 min Proteinase K treatment and 10 min heat inactivation with RNAse inhibitor) to serve as a benchmark for comparison (Figure S1, Supporting Information). The efficiency of lysis and subsequent RNA extraction was quantitatively assessed by measuring the concentration of obtained nucleic acids (ng µL^−1^) and the mean quantification cycle (*C*q) values in a real‐time PCR setup using the glyceraldehyde‐3‐phosphate dehydrogenase (*GAPDH)* gene (Figure S1, Supporting Information). The experiments yielded a range of nucleic acid concentrations and *C*q values, with 30 min Proteinase K lysis and 10 min heat inactivation with RNAse inhibitor showing comparable values to the tube control (Figure S1, Supporting Information).

To demonstrate the optimized protocol, we first dispensed 100 SU‐DHL‐4 cells in an initial volume of 150 nL and performed step 1 and 2 of the protocol (Figure 2; and Figure S2, Supporting Information). We then collected sscDNA from 4 (400 cells), 2 (200 cells), and a single (100 cells) droplets into the tubes and performed steps 4–6 (Figure 2; and Figure S2, Supporting Information). We obtained from about 7000 and 1500 pg µL^−1^ of dscDNA from 400 and 100 cells, respectively (Figure S3E, Supporting Information). We then assessed the quality of the dscDNA using capillary gel electrophoresis, PCR and qPCR. As shown in Figure 2B, we obtained the expected size distribution of dscDNA, from about 300 and 1000 bp, as shown by the electrophoresis results using the high sensitivity Agilent 2100 Bioanalyzer (Figure 2B). Next, we demonstrated successful amplification of short fragment (112 bp) of *GAPDH* and long fragment (1045 bp) of ACTB (*β‐actin*) genes, indicating intact cDNA obtained from the DMA chip (Figure 2C–E). The qPCR analysis of the relative gene expression of *GAPDH* gene showed a statistically significant proportional increase from 100 to 400 cells (Figure 2F). Importantly, minimal spot‐to‐spot variability in the relative expression of *GAPDH* was observed, as indicated by the standard error of the mean for each group, indicating robustness and reproducibility of the developed protocol (Figure 2F). As a next step, we aimed to check the sensitivity of our method and performed the same analysis using only a single cell. As shown in Figure 2B,E, we were able to detect dscDNA obtained from a single cell by capillary electrophoresis and successfully amplify *GAPDH* and *ACTB* genes by PCR (Figure 2A,B,E). Thus, our results show the accuracy and sensitivity of the developed method and demonstrate the successful generation of high‐quality cDNA suitable for various downstream DGEA methods, including qPCR and potentially next generation sequencing (NGS).

### 2.3 Toward Drug‐Induced DGEA from DMA Chip: SU‐DHL‐4 Cells

To demonstrate the application of the established DMA protocol for drug‐induced DGEA, we have characterized the expression of the genes *SYK* (Spleen Tyrosine Kinase) and *GADD45β* (Growth Arrest and DNA Damage‐inducible, beta) following treatment with a cytotoxic anticancer drug on a DMA chip. Both genes play an important role in the regulation of cell survival and apoptosis in CLL, and their study is essential to elucidate the molecular mechanisms driving CLL carcinogenesis, identify potential therapeutic targets, and improve prognostic assessment.^[^ ^44^, ^45^, ^46^, ^47^ ^]^ Understanding the role of *SYK* in aberrant B‐cell receptor signaling and *GADD45β* in stress response and DNA damage repair provides valuable insights that will guide the development of targeted therapies and advance personalized treatment strategies in CLL. The study by Baudot et al. sheds light on the role of *SYK* in coordinating survival pathways in CLL cells, particularly through mechanisms that modulate the expression of Mcl‐1, an essential antiapoptotic protein.^[^ ^44^ ^]^ In addition, research by Woyach et al. highlights the importance of B‐cell receptor signaling in CLL, with *SYK* emerging as a key component of this pathway and a promising therapeutic target,^[^ ^48^ ^]^ and research by Salvador et al. elucidates the role of *GADD45β* in cellular stress responses, including DNA damage repair, which has important implications in the context of CLL carcinogenesis and therapeutic response.^[^ ^46^, ^47^ ^]^

**Figure 3:**
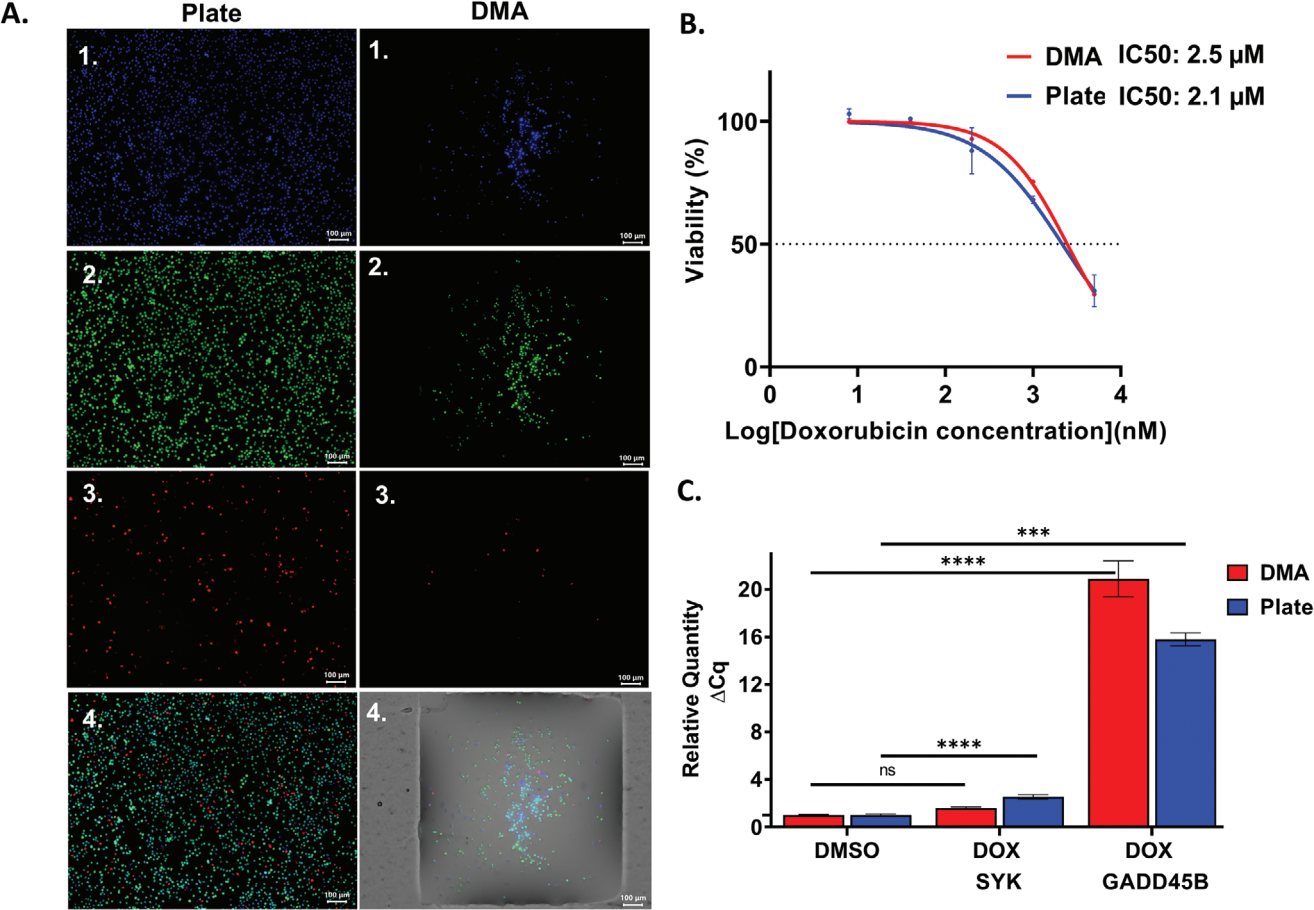
Comparative analysis of SU‐DHL‐4 cell responses to doxorubicin (DOX) treatment in 384‐well plate and on 672‐spot DMA slide. A) Representative images of SU‐DHL‐4 cells stained using live/dead fluorescent staining on both platform plates and the DMA in DMSO control. The staining includes 1) Hoechst, 2) Calcein, and 3) PI. 4) An overlay of the three channels. Images were captured using the Keyence BZ‐X810 microscope. B) Graph showing dose‐response of SU‐DHL‐4 cells to doxorubicin and estimated IC50 values obtained from DMA and 384‐well plate. C) Relative gene expression analysis of *SYK* and *GADD45β* genes in cells exposed to a vehicle control (DMSO) and 1 µm of DOX. Error bars represent the mean ± SEM from three technical repeats (*n* = 3). The bar graphs illustrating the relative gene expression of *SYK* and *GADD45β* from the samples. Statistical significance was determined using one‐way ordinary ANOVA, with highly significant differences ****P < 0.0001, ***P < 0.001, *P < 0.01 for comparison between consecutive groups, “NS” indicates nonsignificant statistical differences between the analyzed groups.

First, we have optimized and validated the protocol for drug‐induced DGEA on DMA using SU‐DHL‐4 cell line with the cytotoxic drug doxorubicin on the DMA chip and in 384‐well plates. For dose‐response assessment, cell viability was assessed by treating cells with doxorubicin over a concentration range from 0.008 to 5 µm for 48 h. Live‐dead fluorescence staining and image‐based analysis were used for evaluation of drug response (**Figure** **3**; and Figure S4, Supporting Information). Figure 3A presents representative images of SU‐DHL‐4 cells stained with live/dead fluorescent dyes on both the 384‐well plates and the DMA platform, with DMSO control after 24‐h incubation. The dose‐response and IC50 values determined for DMA were comparable to the dose‐response obtained from 384‐well plates, being 2.1 and 2.5 µm for plates and DMA, respectively (Figure 3B). We then generated cDNA samples from cells treated with 1 µm doxorubicin on DMA and plates to assess the relative gene expression of *SYK* and *GADD45β* genes. To verify the presence of cDNA in our sample preparation within the droplets, we detected amplification of the housekeeping gene *GAPDH* (Figure S4B,C, Supporting Information). Afterward, the relative gene expression of *SYK* and *GADD45β* genes was analyzed by qPCR as shown in Figure 3C. We have shown an upregulation of both genes in response to doxorubicin treatment in SU‐DHL‐4 cells (Figure 3C). The results obtained on the DMA chip were comparable to those obtained in the 384‐well plate. Treatment with doxorubicin lead to a significant upregulation of both *SYK* and *GADD45β* genes in both DMA and well plate. Specifically, *SYK* expression was upregulated 1.5‐fold in DMA and twofold in plate, while the *GADD45β* gene shows a significant upregulation in response to doxorubicin, with a 20‐fold increase in DMA and a 15‐fold increase in plate. Thus, we have demonstrated the successful adaptation and reliable performance of the drug‐induced DGEA protocol on the DMA platform using the SU‐DHL‐4 cell line. The results obtained from both DMA and conventional 384‐well plates were highly comparable, validating the efficacy of our methodology for high‐throughput drug screening applications.

### 2.4 Toward Drug‐Induced DGEA from the DMA Chip: Patient‐Derived CLL Cells

To demonstrate the applicability of the developed protocol for use with patient‐derived cancer cells, we used CLL cells obtained from patient blood biopsies. In vitro testing of CLL cells for sensitivity to anticancer drugs is an important application that shows good correlation with patient response.^[^ ^49^ ^]^ DGEA is applied to CLL to assess gene expression changes resulting from drug treatment, providing insight into the molecular mechanisms underlying drug response and identifying potential therapeutic targets in CLL. In this study, we aim to demonstrate the feasibility of performing drug‐induced DGEA on patient‐derived CLL cells from the DMA chip, where drug screening and sscDNA preparation are performed on the DMA chip in a nanoliter format.

In our laboratory, we have shown that CLL cells can be cultured and tested on DMA chips and that the viability of CLL cells is higher and more stable when cultured in hydrogel pads on DMA instead of liquid media (manuscript in preparation). Therefore, we cultured CLL in 150 nL of commercially available dextran‐based hydrogel (Cellendes) with 150 nL of medium on top of each hydrogel pad. Therefore, we first adapted the protocol for sscDNA sample preparation using SU‐DHL‐4 cells in 150 nL hydrogel pads on DMA (Figure S5, Supporting Information), successfully demonstrating the expected size distribution of obtained cDNA, spanning between 300 and 1000 bp and amplification of *GAPDH* housekeeping gene (Figure S5, Supporting Information).

**Figure 4:**
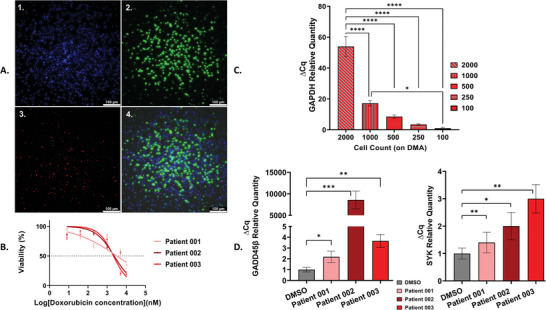
Evaluating viability, cDNA quantity, and Gene Expression of 3 Patients ‐derived CLL Cells on DMA slide. A) Representative mages of CLL patient‐derived cells (patient 003) following 24 h of incubation with 1 µm doxorubicin (DOX) on a DMA slide, stained using live/dead fluorescent staining. The images displayed are: 1) Hoechst, 2) Calcein‐AM, 3) PI, and 4) overlay of the three channels. Images were captured using the Leica Thunder 3D Imager. B) Quantification of GAPDH gene expression in CLL patient‐derived cells (patient 003) by qPCR at different cell numbers ranging from 2000 to 100 cells. B) Comparison of IC50 viability percentages for three different CLL patient‐derived cells indicating their drug sensitivity to DOX treatment. C) Analysis of *SYK* gene expression by qPCR in CLL cells treated with 1 µM DOX. D) Analysis of *GADD45β* gene expression. Statistical analysis showed significant differences (*p* < 0.05) between cell counts and sample preparation methods. Error bars represent the mean ± SEM from three technical repeats (*n* = 3).

Having optimized the protocol for DGEA on hydrogel pads on the DMA chip, we proceeded to test this protocol on patient‐derived CLL cells. First, we tested obtaining cDNA from different numbers of cells ranging from 100 to 2000 cells (**Figure** **4**A–D). Using qPCR analysis, we observed a consistent decrease in *GAPDH* expression with decreasing numbers of primary cells both in tubes and on the DMA chip (Figure 4C; and Figure S7, Supporting Information). This assessment was performed on samples from three different patients, ensuring the robustness of the protocol across different CLL patient samples (Figure S7, Supporting Information). Next, we analyzed the expression levels of the *SYK* and *GADD45β* genes in CLL cells obtained from three patients after treatment with doxorubicin on DMA slide. The IC50 values for doxorubicin in the three different patients were 3.209, 3.109, and 2.141 µm, respectively (Figure 4B; and Figure S6, Supporting Information). We then exposed CLL cells to 1 µm doxorubicin in 150 nL hydrogel pads for 24 h, as represented in Figure 4A. Afterward, cells were subjected to the protocol for cDNA generation. In all three CLL patient‐derived samples tested, relative gene expression analysis revealed upregulation of the *SYK* and *GADD45β* genes, as shown in Figure 4D; and Figure S8 (Supporting Information). In our study, we observed different patterns of gene expression for both *SYK* and *GADD45β* in the different patient samples when compared to the control (DMSO). Patient 1 showed a 1.4‐fold upregulation of *SYK*, indicating a modest increase in expression. In contrast, patient 002 showed a more significant increase with a twofold increase while patient 003 showed an even higher upregulation with a fold increase. These results indicate that the level of *SYK* expression varies between patients, suggesting potential heterogeneity in molecular responses with potential clinical implications. Our observations showed that the degree of up‐regulation in the relative amount of the *GADD45β* gene varied between the patient samples. Specifically, patient 1 showed a remarkable upregulation with a relative abundance of 2.2‐fold. In contrast, patient 002 showed a dramatic upregulation of 8000‐fold, while patient 003 showed a moderate increase of 3.5‐fold compared to the DMSO control. These results underline the existence of patient‐specific responses to the experimental conditions and highlight the need for tailored therapeutic strategies to target *GADD45β* expression. In conclusion, our study reveals a consistent upregulation of *SYK* and *GADD45β* genes in patient‐derived CLL cells treated with doxorubicin, highlighting the potential importance of these molecular changes in the context of therapeutic responses and providing valuable insights for future investigations into CLL treatment strategies. These results demonstrate the feasibility of performing such protocols on patient‐derived cells, thereby facilitating the exploration of personalized medicine approaches to CLL treatment.

To date, no previous research has investigated the direct effect of the drug doxorubicin on the *SYK* and *GADD45β* genes in the SU‐DHL‐4 cell line or CLL patient‐derived cells. However, our results are consistent with previous research showing that DNA damage in cancer cells induced by chemical inhibitors of *SYK* leads to an upregulation of *SYK* expression, accompanied by an increase in p53 expression in the HCT116 and HT1080 cell lines.^[^ ^50^ ^]^ According to Wiest et al., the *GADD45* family of proteins, consisting of *Gadd45a*, *Gadd45β*, and *Gadd45g*, serve as stress sensors, particularly in response to oncogenic stress, and regulate various cellular processes, such as cell cycle, DNA replication/repair, and survival through interactions with other proteins.^[^ ^51^ ^]^ Guo et al. demonstrated the effects of *GADD45g* overexpression, showing its role in inducing apoptosis, differentiation, growth inhibition, and enhancing chemosensitivity in primary leukemia cells from patients with AML.^[^ ^47^ ^]^Our findings are consistent with previous research and support the involvement of *GADD45β* in stress response and DNA damage repair, as evidenced by its significant upregulation in response to doxorubicin treatment in both DMA and plate platforms.

## 3 Conclusion

In conclusion, our study presents a novel approach to drug‐induced DGEA that addresses the challenges associated with high‐throughput screening and limited cell availability, particularly in the context of patient‐derived cancer cells. We have developed a streamlined protocol for DGEA that significantly reduces the amount of reagents required and enables the analysis of minute number of unique patient‐derived cells on the DMA platform. We demonstrate the implementation of this protocol on both cell lines and primary patient‐derived CLL cells, demonstrating its adaptability and relevance in clinical settings. Importantly, our results show comparative and correlative results between the DMA platform and conventional methods, highlighting the reliability and accuracy of our approach.

Furthermore, we provide evidence for the efficacy of the platform by identifying upregulation of key genes, *SYK*, and *GADD45β*, following the treatment of CLL cells with the drug doxorubicin. These results highlight the utility of our optimized protocol in revealing molecular changes associated with drug response, thereby advancing our understanding of cancer biology and facilitating personalized therapeutic strategies in precision oncology. Thus, our research not only introduces a novel methodology for drug‐induced DGEA in a miniaturized format, but also highlights its potential for broader applications in a high‐throughput manner. The use of a 672‐DMA drug library for precision oncology opens the horizon for high‐throughput drug‐induced DGEA in nanoliter formats from primary patient‐derived cells, providing a method for in‐depth analysis of molecular changes in unique patient‐derived cells upon drug treatment.

Following the comparison to the tube protocol, the DMA protocol shows a significant reduction in cell lysis volume from 2.25 to 0.2 µL, a reduction of ≈91%. Similarly, for the reverse transcription mix, the volume used per reaction is reduced from 5.1 to 0.15 µL (150 nL per spot), resulting in a reduction in reagent consumption across the platforms by ≈97.06%. We have also significantly reduced the amount of drug required. In this study, doxorubicin was dispensed onto 672 DMA slides at a volume of 1.5 nL per spot, reducing drug consumption by ≈99% compared to the 384‐well plate where the same drug concentrations were prepared at volumes of 2.5 µL per well. In addition, we have significantly reduced the number of cells used. While the 384‐well plate used 10000 SU‐DHL‐4 cells per well in 22.5 µL of medium, the DMA chip used only 100 SU‐DHL‐4 cells in medium and 2000 CLL cells in hydrogel per spot, each requiring only 150 nL of volume. Our results highlight the potential of the DMA chip to perform comprehensive DGEA analysis in a nanoliter format comparable to plates.

To address the issue of tissue heterogeneity, particularly in the context of blood cancers, we took several key steps to ensure the reliability of our analysis. First, we focused on achieving high purity in the patient‐derived CLL cells, with the samples containing high percentage of B lymphoma cells, which are the primary targets of our study. By isolating and analyzing these cancer cells, we aimed to minimize the influence of nonmalignant cell populations and gain a clearer understanding of the characteristics and behavior of the CLL cells.^[^ ^49^ ^]^ It is important to note that in blood cancers, unlike in solid tumors, cells from the entire biopsy are mixed together, which means that ideally, all cell types should be represented in each droplet. However, as the cell number decreases, there is an increased risk that some cell types may not be adequately represented. While this is a challenge inherent to biopsies, it is a common issue across all types of cancer research, as biopsies never capture every cell type within the tumor. Regarding future approaches, we recognize the potential value of single‐cell analysis. Although our current study focuses on qPCR from populations of cells, combining this with automated droplet collection opens the possibility of performing single‐cell RNA sequencing after treatment. While we do not commit to this approach in the present study, it is a feasible option for future investigations to further investigate cellular heterogeneity.

As a future perspective, the successful validation of DGEA with a single drug paves the way for a wide range of applications, including drug screening in other primary cell types using clinically relevant drug panels and candidates. This approach can also be extended to test important CLL drugs, such as the BCL‐2 inhibitor venetoclax, the anti‐CD20 monoclonal antibody obinutuzumab, and BTK inhibitors like ibrutinib, acalabrutinib, and zanubrutinib. Additionally, our recent work has shown the feasibility of automated droplet collection and transfer using the ANDeS device, opening the door to high‐throughput drug screening and gene expression analysis on the DMA chip. The established protocol here can be further expanded from qPCR to mRNA sequencing achieving integration of NGS into our platform (manuscript in preparation). This development significantly broadens the scope of DGEA on DMA, making it a powerful tool for extracting unique insights from patient samples regarding drug response. The protocol demonstrated in this study underscores the great potential of DGEA on the DMA chip.

## 4 Experimental Section

### Reagents and Equipment

The SU‐DHL‐4 cell line was purchased from ATCC CRL‐2957 (USA). Roswell Park Memorial Institute (RPMI) 1640 medium, fetal bovine serum (FBS), and penicillin‐streptomycin (P/S), Dulbecco's phosphate‐buffered saline (DPBS), trypan blue solution 0.4%, Maxima H minus reverse transcriptase, SYBR Safe DNA gel stain, Hoechst, Calcein AM, propidium iodide (PI) were purchased from Thermo Fisher Scientific (Germany). Human serum (HS) was purchased from Sigma‐Aldrich (USA). Exonuclease I was purchased from VWR International GmbH (Germany). Customized forward (F) & reverse (R) primers for Glyceraldehyde‐3‐phosphate dehydrogenase (*GAPDH*), ACTB *(β‐actin*), spleen‐associated tyrosine kinase (*SYK*), growth arrest, and DNA damage inducible beta (*GADD45β*) genes were purchased from Integrated DNA Technologies (IDT) (Belgium). Doxorubicin was purchased from StemCell Technologies (Germany). Ampure XP beads were purchased from Beckman Coulter (Germany) and nuclease‐free water (NFW) was purchased from Life Technologies GmbH (Germany). Gel electrophoresis was performed using 1 kb DNA ladder, low molecular weight DNA ladder and gel loading dyes was purchased from New England BioLabs GmbH (Germany). The Taq PCR Master Mix Kit was purchased from Qiagen GmbH (Germany), and the Gotaq qPCR Master Mix was purchased from Promega GmbH (Germany). 3‐D Life Dextran‐PEG Hydrogel SG was purchased from Cellendes GmbH (Germany). 672‐spot DMA slides (Cat. Nos. G‐np‐102) were purchased from Aquarray GmbH (Germany). Additional materials included a parafilm roll from Fischer Scientific GmbH, a metal in situ adapter from Antylia Scientific, Cole‐Parmer GmbH, a 3D printed PCR chamber lid designed using Rhinoceros 3D software and manufactured by Creabis GmbH, and a neodymium block magnet purchased from Supermagnete Webcraft GmbH (Germany). Standard polystyrene Petri dishes, PCR microtubes and 40 µm sieves were purchased from Greiner Bio‐One GmbH (Germany). 384‐well µL plates were purchased from Axygen Scientific GmbH (Germany). Cell counting was performed using a Life Technologies Countess II cell counter and a Thoma cell counting chamber. Microscopy was performed using a Leica Thunder 3D imager and a Keyence‐BZ‐X810 microscope. An I‐DOT ONE liquid dispenser with integrated humidifier from Dispendix GmbH (Germany) was used for dispensing cells on DMA. The drug doxorubicin was printed at different concentrations using the liquid dispenser sciFLEXARRAYER S11 from Scienion (Germany).

### Patient‐Derived CLL Cells

Peripheral blood samples were obtained from patients diagnosed with CLL. The samples were obtained from the Molecular Therapy in Hematology and Oncology and the Department of Translational Oncology at the National Centre for Tumour Diseases and the German Cancer Research Centre in Heidelberg, Germany. After blood collection, Ficoll gradient separation (GE Healthcare, Munich, Germany) was performed, and the mononuclear cells obtained were cryopreserved. All protocols regarding the collection and preservation of patient‐derived cells were approved by the Heidelberg Ethics Committee (University of Heidelberg, Germany; S‐356/2013).

### Cell Culture

SU‐DHL‐4 cells were cultured in RPMI 1640 medium supplemented with 10% v/v FBS and 1% v/v P/S. Cell culture wфы maintained in a standard cell culture incubator at 37 °C with 5% CO_2_. All experiments were performed with ≈95% cell viability.

Patient‐derived CLL cells were obtained from the University of Heidelberg and stored in liquid nitrogen. To thaw CLL cells, the vial containing frozen cells was placed in a 37 °C water bath for 2–3 min to ensure rapid thawing of cells. The cell suspension was then immediately transferred to RPMI 1640 medium containing 10% heat‐inactivated FBS and 1% P/S. After centrifugation at 400 g for 5 min, the cell pellet was resuspended in the same cell culture medium and then filtered through a 40 µm strainer. The filtered CLL cells in medium were again centrifuged at 400 g for 5 min. Finally, the resulting cell pellet was resuspended in RPMI 1640 medium supplemented with 10% heat‐inactivated HS and 1% P/S. The cells were then printed on DMA slides at the desired concentrations.

For culturing cells on DMA chips in liquid media, cells were first counted, diluted to the desired cell density (to obtain a specific number of cells per spot) and dispensed onto a sterile 672‐spot DMA slide using an I‐DOT ONE automated dispenser. The DMA slide was sterilized with 100% ethanol and dried under a sterile bench for 10 min. A humidified Petri dish (10 cm) was prepared to maintain humidity and prevent droplet evaporation on the DMA slide. This humidified Petri dish was prepared by placing a tissue pad in the lid, wetting it with 6 mL PBS, and adding 2 mL PBS inside the Petri dish. The humidified Petri dish was prepared in advance and placed in a cell culture incubator to equilibrate the humidity. After dispensing, the DMA chip containing cells was immediately placed in the humidified Petri dish and placed in a standard cell culture incubator.

For the culture of CLL cells on DMA chips in hydrogels, Cellendes 3‐D Life Dextran‐PEG Hydrogel SG Kit was used. The hydrogel mixture was prepared according to the manufacturer's instructions (Table S7, Supporting Information). The cell density in the cell suspension added to the mixture was adjusted according to the desired number of cells per spot. The hydrogel mixture was then immediately dispensed onto DMA slides at a volume of 150 nL hydrogel per spot. The DMA slide was then transferred to a humidified Petri dish and placed in a cell culture incubator to allow the gel to solidify for at least 45 min. After solidification, a total of 150 nL of cell culture medium (RPMI 1640 medium supplemented with 10% heat‐inactivated HS and 1% P/S) was added to the established hydrogel pads and returned to the humidified Petri dish and cell culture incubator for culture.

### Sample Preparation and cDNA Generation on DMA Chips

Before starting the experiments, it is essential to sterilize the clean bench with RNASeZap and to work in an aseptic environment. All reagents used in this experiment were thawed on ice unless otherwise specified in the kit manufacturer's protocols. The experimental procedure for the experiment is shown in Figure 2A. To prevent evaporation during sample preparation, water droplets were spotted around the droplets with the experiment (4, 2, or 1 droplets), leaving 1 row of droplets empty around the sample to simplify manual collection of the droplets (Figure S2, Supporting Information). Then 100 nL of the cell suspension with the desired cell concentration was printed in four, two, or one droplets according to the scheme (Figure S2, Supporting Information). Images were captured using Keyence‐BZ‐X810 to verify the number of cells per spot prior to lysis (Figure S3A–D, Supporting Information). The method of limited dilution was used for obtaining a single cell per droplet. The presence of a single cell in the droplets was verified by microscopy. Once the positions of the single cells were identified, a protocol was created on the I‐DOT One to dispense reagents only into the droplets containing single cell. Subsequently, 100 nL of cell lysis buffer containing Proteinase K (Table S2, Supporting Information) was added to each droplet with single cell, followed by the addition of 10 nL of E3V6NEXT primer (2 µm). The DMA slide was then transferred to a PCR humidity chamber. A PCR humidity chamber was assembled with a metal adapter for a 96‐well thermocycler (Bio‐Rad Laboratories GmbH, Germany).^[^ ^20^ ^]^ Tissue pads were cut into strips and placed around the edges to allow space for a DMA slide. These pads were moistened with a total of 4 mL of water. The chamber was sealed with a custom 3D‐printed lid made of heat‐resistant polyamide to maintain adequate humidity during the temperature steps. This chamber containing the DMA slide was placed in the thermocycler for a 15‐min for Proteinase K digestion at 50 °C, followed by a 12‐min heat inactivation step at 80 °C. During the cell lysis process, the reverse transcription (RT) mix was prepared according to Table S3 (Supporting Information). Upon completion of the cell lysis process, the PCR humidification chamber containing the DMA slide was placed on ice for 2 min. Cell lysis was checked under the microscope (Figure S3A–D, Supporting Information). Subsequently, 150 nL of reaction mix was added per spot. The DMA slide was then placed in a PCR thermocycler for conversion of mRNA to cDNA, which was performed at 42 °C for 100 min. Upon completion of the reverse transcription step, the PCR humidity chamber was placed on ice for 2 min. Next, 10 µL of water was pipetted onto each experimental droplets (4, 2, or 1) to manually collect the cDNA samples from the DMA and transfer them to PCR microtubes. The single‐stranded cDNA (ss‐cDNA) was then purified using AMPure XP beads according to the Agencourt AMPure XP manufacturing instructions. After purification, excess of primers, salts, enzymes, and nucleotides were removed by a washing procedure, and then ss‐cDNA was subjected to exonuclease treatment with exonuclease I at 37 °C for 20 min, followed by a heat inactivation step at 80 °C for 10 min (Table S4, Supporting Information). The cDNA amplification mix (Table S5, Supporting Information) was then added to the exonuclease I digested samples. sscDNA was amplified using the following settings: initial denaturation at 98 °C for 3 min, followed by 21 cycles of denaturation at 98 °C for 15 s, annealing at 65 °C for 30 s, extension at 68 °C for 4 min, and a final extension at 72 °C for 10 min. The samples were then cooled at 8 °C and stored at −20 °C. The concentration of ds‐cDNA was measured using a Nanodrop 2000 UV–vis spectrophotometer (Thermo Fisher Scientific) and quantified by absorbance measurements and 260/280 and 260/230 ratio analysis. Finally, the complete double‐stranded cDNA (dscDNA) was purified with AMpure XP beads using the same procedure as for sscDNA. The resulting dscDNA was checked for quality using standard PCR and gel electrophoresis, RT‐PCR and bioanalysis to confirm correct sample preparation on the DMA platform.

For sample preparation in hydrogel arrays, an additional lysis step was introduced to ensure complete lysis of the cells within the hydrogels. Lysis was performed in a thermocycler for 15 min at 50 °C, followed by 12 min of proteinase K inactivation at 80 °C. The lysis step was repeated by adding 100 nL of lysis solution, followed by 15 min at 50 °C and 12 min at 80 °C. After the lysis step, the protocol was carried out as previously described for liquid medium.

### Drug Treatment

Doxorubicin was administered at the following concentrations: 5, 1, 0.2, 0.04, and 0.008 µm (Table S1, Supporting Information). It was dispensed onto 672 DMA slides using the Scienion sciFLEXARRAYER S11 liquid dispenser (Germany). For 384‐well plate treatment, the same drug concentrations were prepared to be pipetted in 2.5 µL volumes per well. 10000 SU‐DHL‐4 cells were used per well in 22.5 µL of medium. For the DMA chip, 100 SU‐DHL‐4 cells in medium and 2000 CLL cells in hydrogel were used per spot in 150 nL volumes. The cells were treated with doxorubicin for 48 h. Cell viability was assessed by live/dead fluorescent staining including Hoechst, Calcein, and PI. IC50 was determined using GraphPad Prism software.

To assess *SYK* and *GADD45β* gene expression levels, cells were exposed to IC50 concentrations of doxorubicin for 48 h, followed by the sample preparation protocol described in “Sample preparation and cDNA generation on DMA chips.”

### PCR and Gel Electrophoresis

Two housekeeping genes, *GAPDH* and *β‐actin*, were selected for quality control of the obtained cDNA. A positive control sample was used together with nuclease‐free water (NFW) as a negative control. Primer sequences for PCR are listed in Table S6 (Supporting Information). Taq PCR Mix (Qiagen, Germany) was used for the PCR reaction and all reagents were thawed on ice. Further details are provided in Table S8 (Supporting Information). The PCR protocol included 35 cycles including an initial denaturation step at 94 °C for 3 min, followed by cycling steps of denaturation at 94 °C for 30 s, annealing at 54 °C for 30 s, and extension at 72 °C for 45 s, followed by a final extension step at 72 °C for 3 min. The reaction was then cooled to 8 °C for an indefinite period. The resulting PCR amplification products were run in 1.5% agarose gel at 100 volts for 75 min in a gel electrophoresis apparatus (Bio‐Rad Laboratories GmbH, Germany). DNA was visualized by staining with SYBR Safe DNA Gel Stain and imaged using a UV transilluminator (Bio‐Rad Laboratories GmbH, Germany).

### Quantitative PCR (qPCR)

A standard manufacturer's protocol of GoTaq qPCR Master Mix was used for qPCR analysis. Gene‐specific primers are listed in Table S6 (Supporting Information). The reaction was performed on a StepOne real‐time PCR system (Life Technologies GmbH, Germany). Gene expression analysis was performed as previously described.^[^ ^52^ ^]^

### Bioanalyzer

The dscDNA samples were first quantified for concentration and purity using a Nanodrop spectrophotometer. Agilent 2100 Bioanalyzer chips were then prepared according to the manufacturer's guidelines. Electropherograms and data reports were generated for each sample condition. The reports provided information on the quality, size distribution, and concentration of the DNA.

### Statistical Analysis

Data were analyzed using GraphPad Prism software. All experiments were performed in triplicate. Error bars represent mean ± SEM. Statistical significance was determined using ordinary one‐way ANOVA, with highly significant differences (****p < 0.0001, ***p < 0.001, **p < 0.01) for comparisons between consecutive groups, while “NS” indicates nonsignificant statistical difference between the data groups.

### Ethical Statement

All experiments were performed in accordance with relevant guidelines and regulations. Human samples used in this study were obtained with the approval of the Ethics Committee of the University of Heidelberg, Germany (S‐356/2013). Written informed consent was obtained from all patients who donated tumor material prior to participation. No patient‐identifying information was disclosed in this study, and the data generated were used solely for protocol optimization and DGEA following drug treatment on the DMA platform, without being linked to individual patient data.

## Conflict of Interest

R.F, S.C, M.Z, M.S, D.T, L.S, M.C, A.‐K. K, and S.D declare no conflict of interest related to the publication of this article. The authors declare the following potential conflict of interest related to the research, authorship, and/or publication of this article: P.A.L. and A.A.P. have been shareholders of Aquarray GmbH since March 2018, in addition to their employment at the Karlsruhe Institute of Technology.

## Supporting information



Supporting Information

## Data Availability

The data that support the findings of this study are available at DOI: https://doi.org/10.35097/e6434y1n86568and
