# Supplementary material for: Drug‐Induced Differential Gene Expression Analysis on Nanoliter Droplet Microarrays: Enabling Tool for Functional Precision Oncology
Source: Adv Healthc Mater. 2024 Oct 23;14(1):2401820. doi: 10.1002/adhm.202401820 (PMC11694083; doi:10.1002/adhm.202401820)
Supplement: Supplementary file 1 — Supporting Information [file ADHM-14-0-s001.docx]

**Supporting Information:**

*
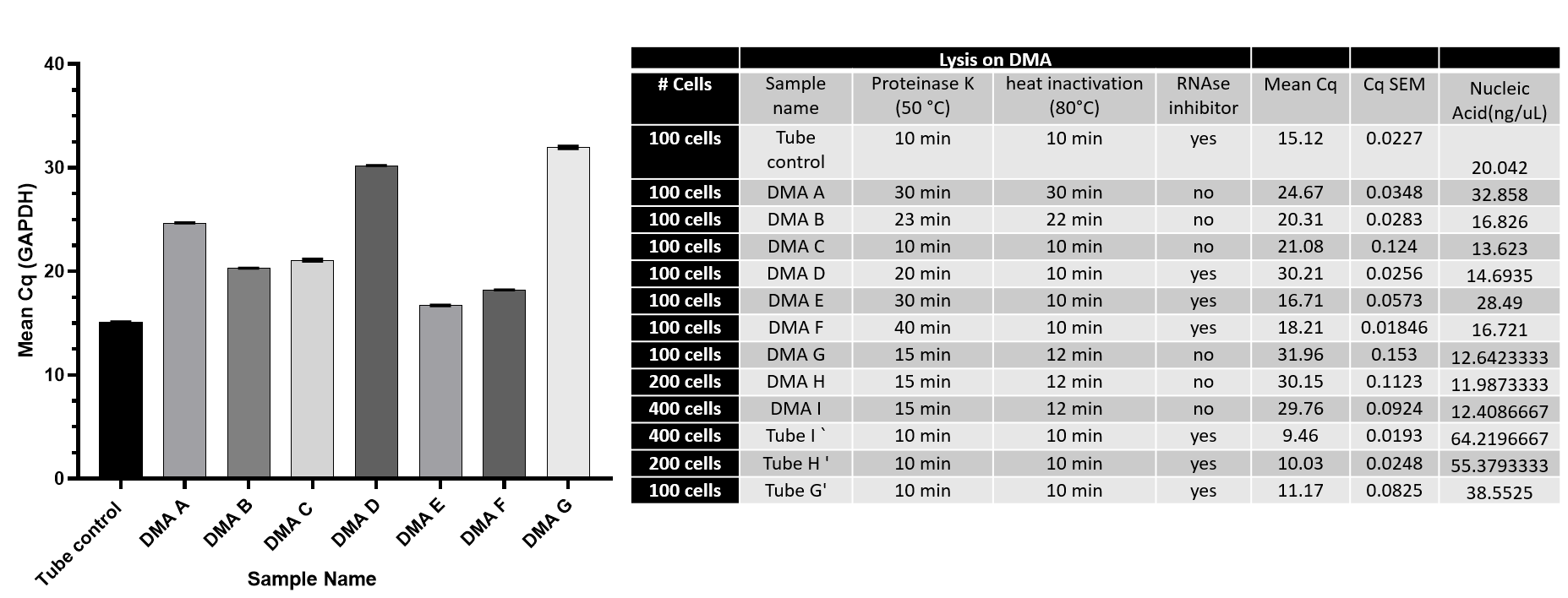
*

**Figure S1.** Mean Cq Values Across Various Conditions: Optimizing Cell Lysis Protocol on DMA. A bar graph illustrates the mean Cq value of the GAPDH housekeeping gene using different samples prepared on the DMA platform with varying conditions. Each sample is prepared in triplicate for technical repeats. Error bars represent the standard error of the mean (SEM)**.**  Error bars represent the mean ± SEM with n = 3.


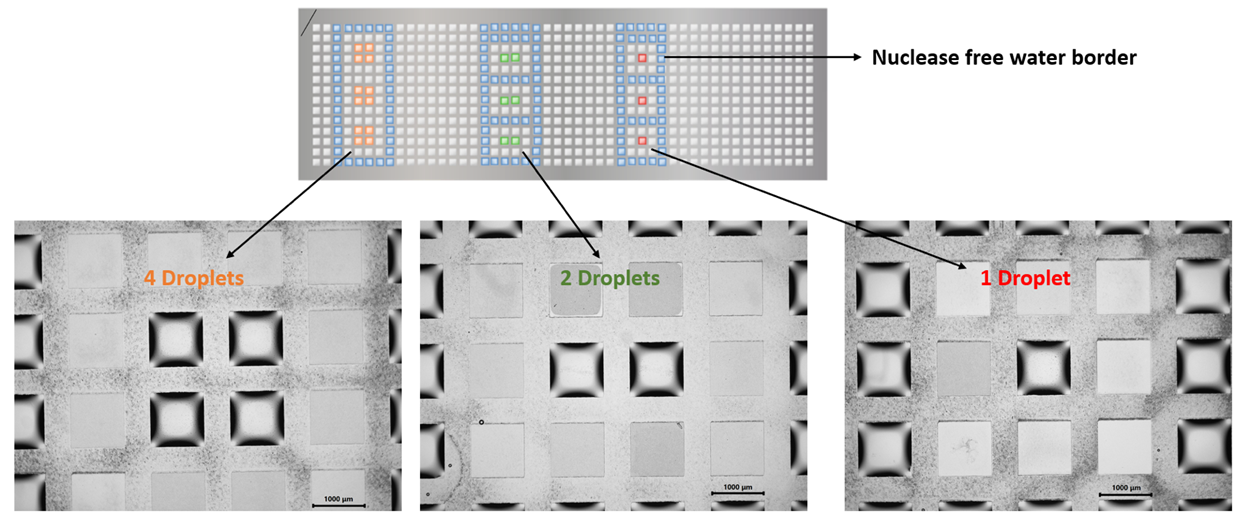


**Figure S2.** DMA slide layout for cell printing. Schematic representation of the pattern layout on a DMA slide, illustrating the arrangement of cells printed in droplets. Each spot contains a total of 100 cells, with 4 droplets equating to 400 cells, 2 droplets equating to 200 cells and 1 droplet equating to 100 cells. Nuclease-free water (NFW) is applied around the SU-DHL-4 cell spot to ensure adequate humidity.

**
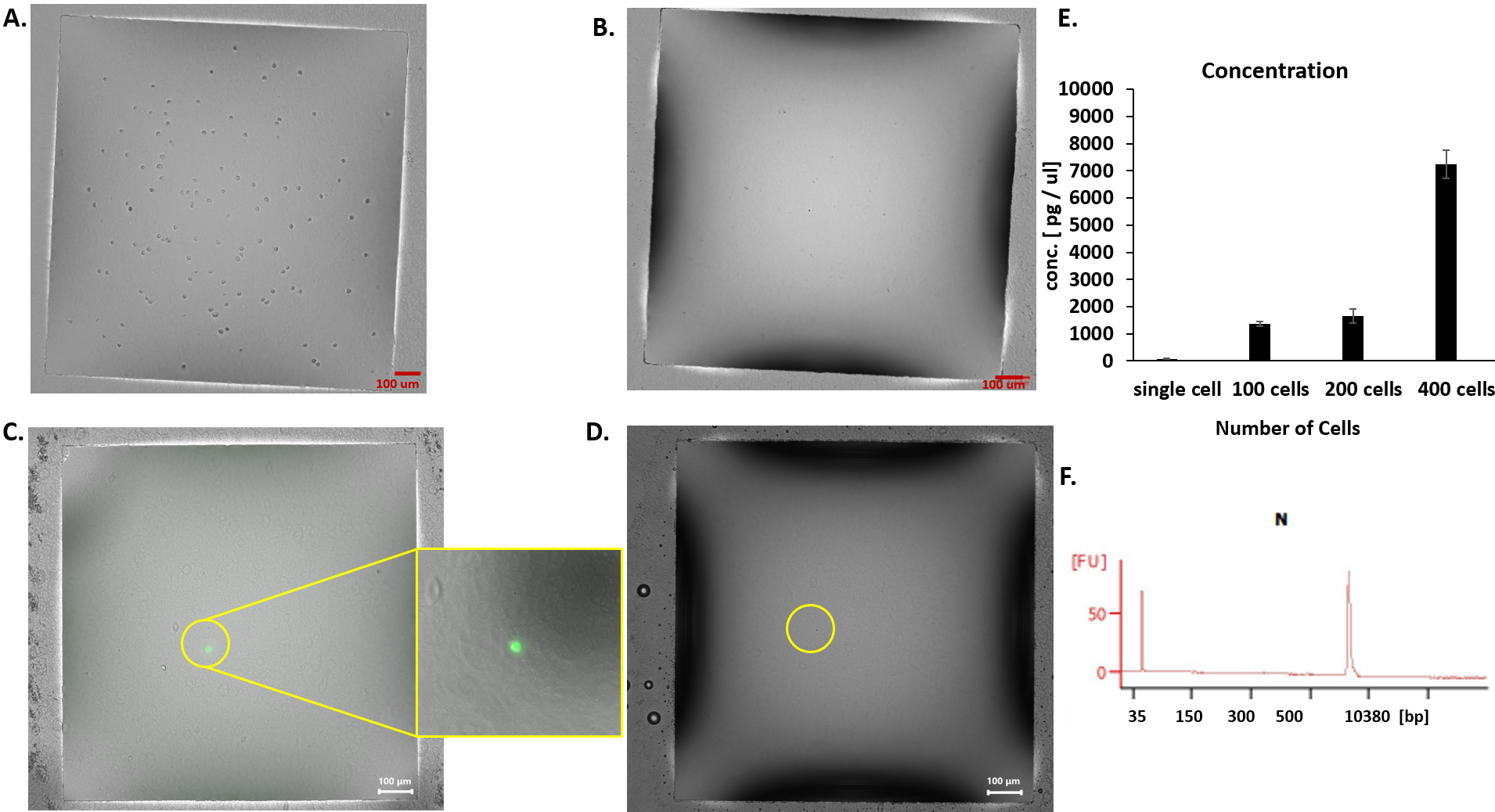
**

**Figure S3.** Cell printing, lysis and cDNA concentration analysis. (A) One hundred SU-DHL-4 cells were printed and checked using a Keyence-BZ-X810 microscope (x10 magnification) before the lysis step. (B) In the same spot, after the lysis step, the one hundred SU-DHL-4 cells were lysed. (C) A single cell, stained with Calcein AM dye, is shown within the center of a 1 mm × 1 mm droplet, as indicated by the yellow circle and magnified 20x. The cell exhibits a fluorescent signal due to the Calcein AM staining. (D) The single cell was then subjected to lysis, and a 10x image was taken post-lysis. (E) A bar graph illustrates the cDNA concentration (pg/µL) as the number of SU-DHL-4 cells used increases, from a single cell to 400 cells. Error bars represent the mean ± SEM with n = 3. (F) An electropherogram shows the Bioanalyzer results of a negative control. No template (no cells) was used during sample preparation.


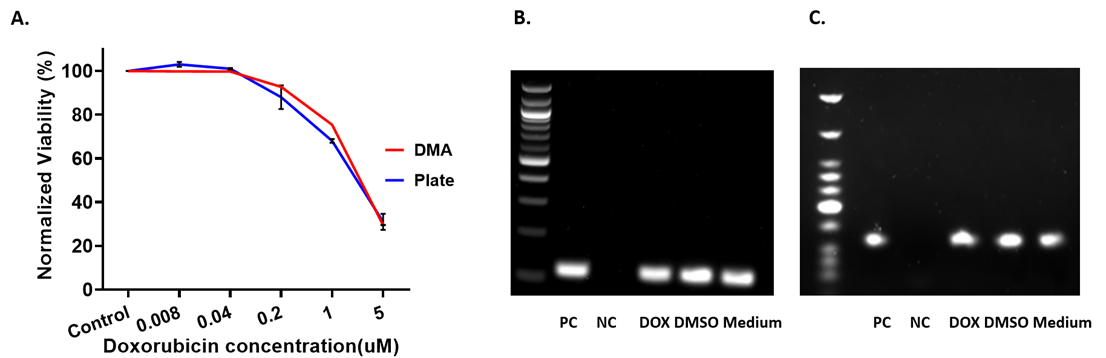


**Figure S4.** Comparative analysis of SU-DHL-4 cell viability and *GAPDH* expression in response to DOX treatment. (A.) Normalized viability (%) of SU-DHL-4 cells after treatment with DOX drug at different concentrations ranging from 5 uM to 0.008 uM. Comparison between DMA vs. plate. Error bars represent the mean ± SEM with n = 3. (B.- C.) Gel electrophoresis analysis of *GAPDH* expression in samples including positive control (PC), negative control (NC), DOX, DMSO and medium from plates and DMA respectively.


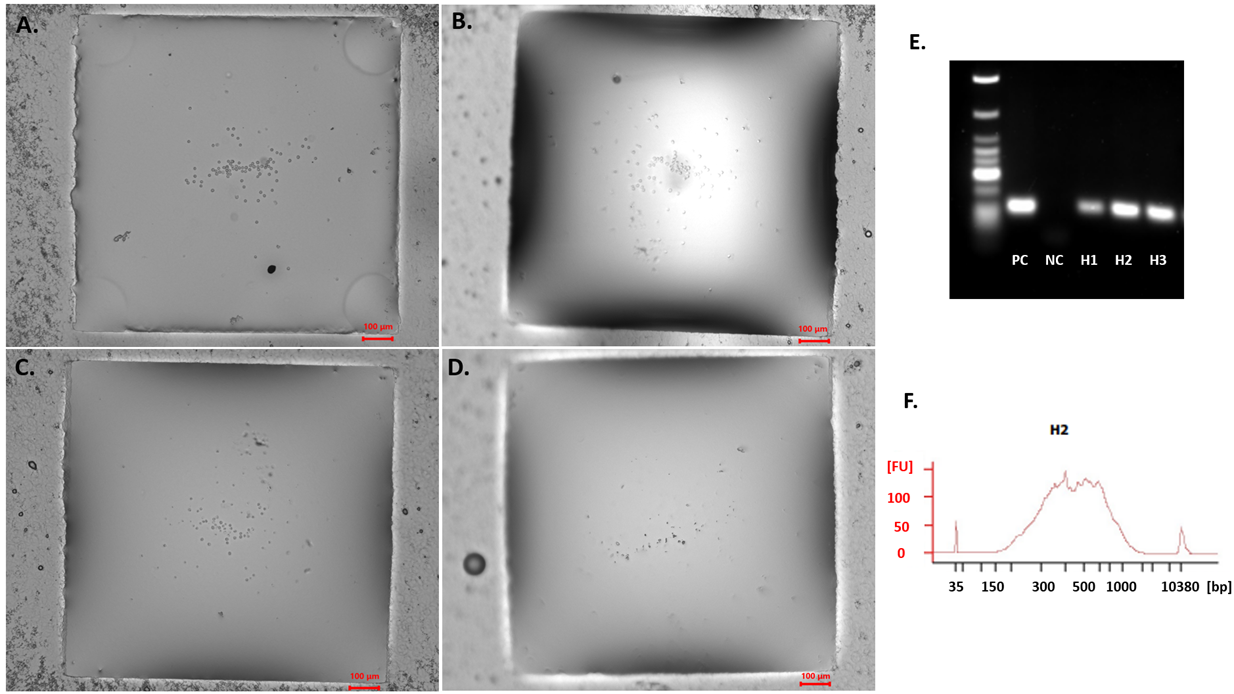
**Figure S5.** Sample preparation in hydrogel on a DMA slide. (A) Representation of 100 cells printed in the hydrogel mixture before solidification, observed under the Keyence-BZ-X810 microscope (x10 magnification). (B) The condition after solidification, followed by printing in RPMI medium for 24 hours incubation. (C) After the initial lysis step, the cell membrane integrity remains relatively rigid. (D) After an additional lysis step, the cells show complete lysis, indicating the loss of cell membrane integrity. (E) Gel electrophoresis results for cDNA synthesized in hydrogel in 3 technical replicates showing the *GAPDH* PCR product. The 'PC' lane represents the positive control, while the 'NC' lane is the negative control without template. Panels (H1), (H2) and (H3) show 3 technical replicates of samples prepared in hydrogel. Lane 1 corresponds to the 1 kb DNA ladder. (F) Qualitative assessment of the synthesized cDNA prepared on the DMA slide using the Agilent 2100 Bioanalyzer with High Sensitivity DNA Analysis Kit.

**
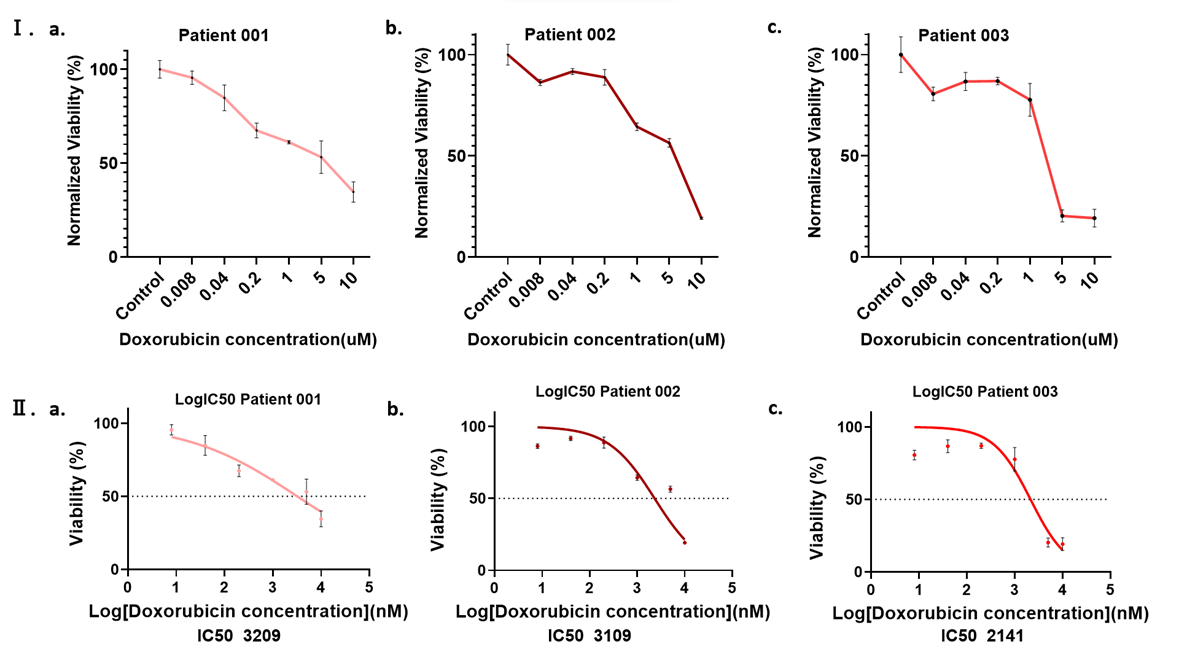
Figure S6.** Comparative evaluation of DOX drug sensitivity for 3 patient-derived CLL cells on DMA slide. (I) Normalized viability evaluation for 3 different patient-derived CLL cells (2,000 cells per spot) treated on the DMA slide with different drug concentrations ranging from 10 uM to 0.008 uM. Panels (a), (b) and (c) represent responses from 3 different CLL patients. (II) IC50 plots for the same three patients, illustrating the concentration at which 50% inhibition of cell viability occurs upon DOX treatment. Subpanels (a), (b) and (c) correspond to individual patient responses and provide insight into the variation in drug sensitivity between CLL patients. Error bars represent the mean ± SEM with n = 3.

**
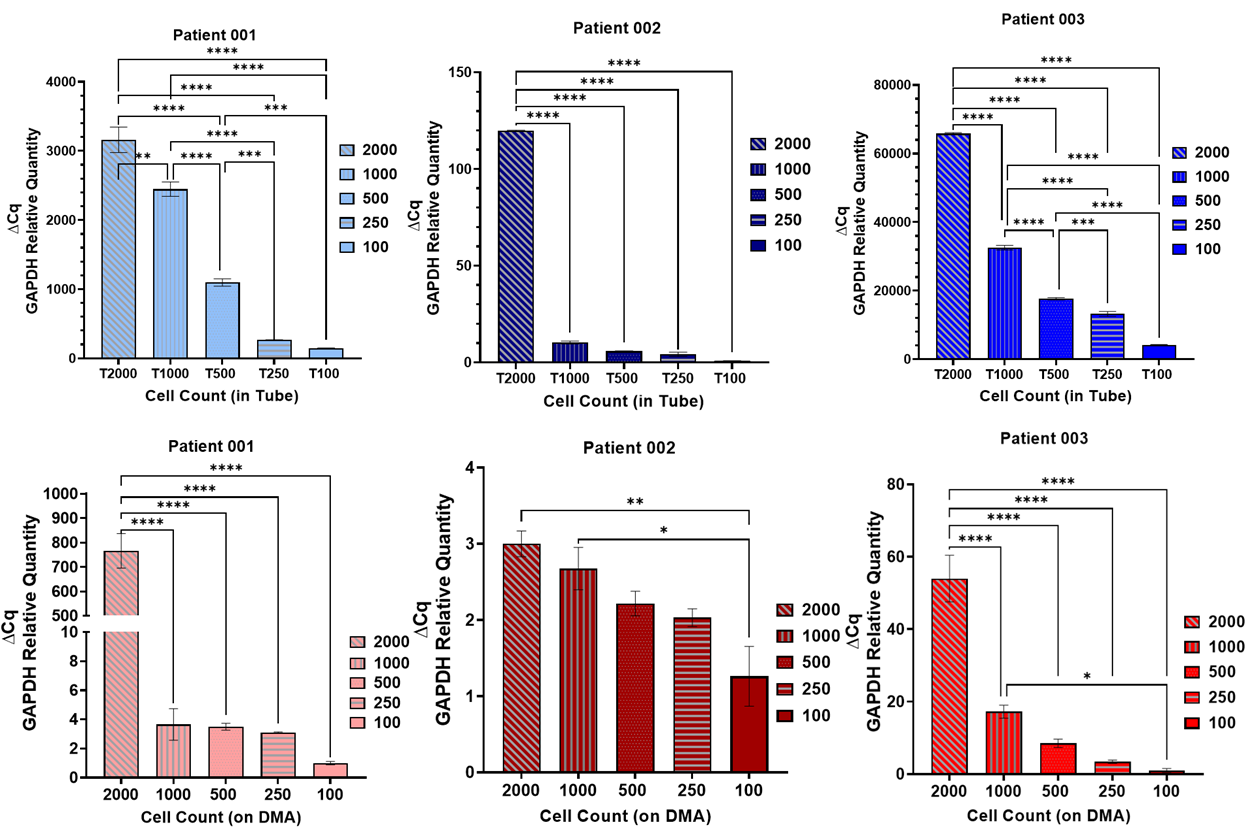
**

**Figure S7.** Evaluation of the relative gene expression of the housekeeping gene GAPDH in different cell counts of CLL patient-derived cells. The plots illustrate the expression levels across different cell counts (2000 cells down to 100 cells) in Tube and similarly on DMA prepared from three different CLL patients. The data provide insight into the effect of cell count and sample preparation method on the relative expression of the housekeeping gene GAPDH. Statistical analysis revealed significant differences (p < 0.05) between cell numbers and sample preparation methods. Error bars represent the mean ± SEM with n = 3.


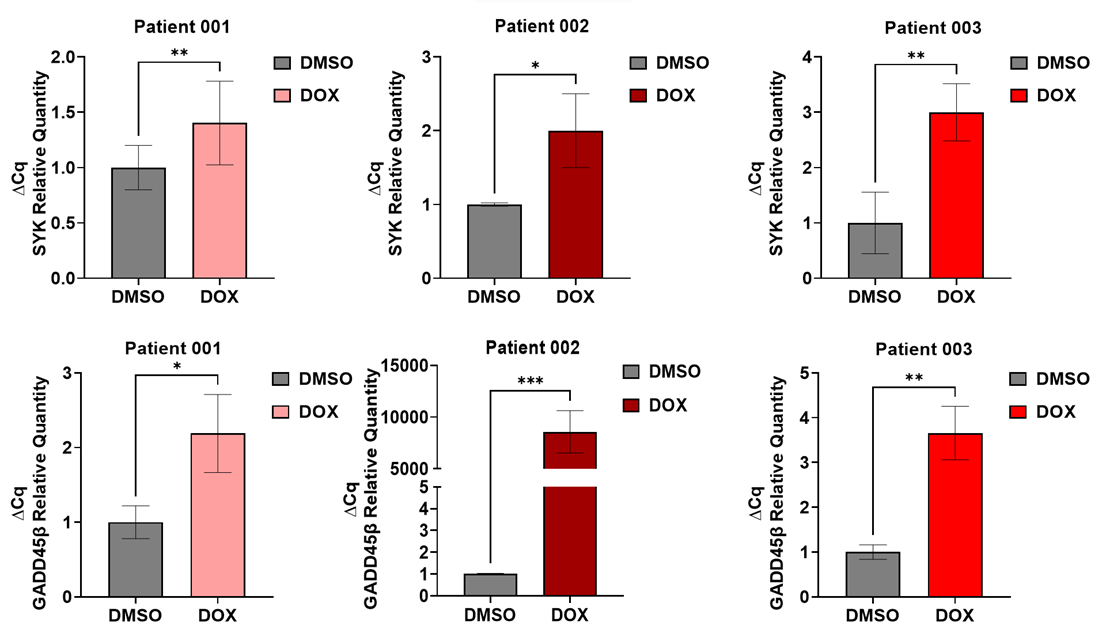


**Figure S8.** Evaluation of relative gene expression of *SYK* and *GADD45β* genes using qPCR. The plots show the effect of 1 uM DOX drug treatment on the expression levels of *SYK* and *GADD45β* genes in CLL cells from three different patients, providing insight into the molecular response of CLL cells to DOX treatment and highlighting potential variations between individual patient samples. Statistical analysis revealed significant differences (p < 0.05) between cell counts and sample preparation methods. Error bars represent the mean ± SEM with n = 3.

| **Drug** | **Target** | **Stock** | **C5** | **C4** | **C3** | **C2** | **C1** |
| --- | --- | --- | --- | --- | --- | --- | --- |
| Doxorubicin  (Final concentration Per well) | Topoisomerase Ⅱ | 1 mM | 5 μM | 1 μM | 0.2 μM | 0.04μM | 0.008μM |

**Table S1.** Proof of concept: Doxorubicin differential drug concentrations.

**Table S2:** Lysis buffer reaction mix:

| **Reagent** | **Volume** |
| --- | --- |
| NEB HF Phusion buffer (5x) | 0.2 µL |
| Nuclease free water | 75 µL |
| Proteinase K (20 mg/mL) | 5.5 µL |

**Table S3:** Reverse transcription reaction mix (RT). During cell lysis, prepare the reverse transcription mix as follows:

| **Reagent** | **Volume** |
| --- | --- |
| Polyethylene glycol 40% solution (v/v) | 2 µL |
| Nuclease free water | 2 µL |
| 5X Maxima H RT buffer | 4 µL |
| 10 mM dNTPs | 0.8 µL |
| E5V6NEXT (100 µM) | 0.4 µL |
| RNaseOut | 0.5 µL |
| Maxima H reverse transcriptase | - 1. µL |

**Table S4** Exonuclease treatment mix. To the 17 µL cDNA, add the following:

| **Reagent** | **Volume** |
| --- | --- |
| 10X Exonuclease I buffer | 2 µL |
| Exonuclease I (20U/µL) | 1 µL |

**Table S5:** cDNA amplification mix. Prepare the PCR master mix as follows:

| **Reagent** | **For 1 Reaction** |
| --- | --- |
| Terra Direct buffer (2x) | 25 µL |
| SINGV6 primer (10 µM) | 1 µL |
| Terra polymerase (1.25 U/µL) | 1 µL |
| Nuclease free water | 3 µL |

**Table S6:** Primers sequences

| **Primer** | **Sequence** |
| --- | --- |
| GAPDH Forward | CTC TGC TCC TCC TGT TCG AC |
| GAPDH Reverse | CCC AAT ACG ACC AAA TCC GT |
| SYK Forward | GGT CCT CAC CAA AGT TCT CTG |
| SYK Reverse | CCA GGT AAT CTT CTG CCT CCT |
| GADD45B Forward | GCC AGG ATC GCC TCA CAG TG |
| GADD45B Reverse | GGA TTT GCA GGG CGA TGT CA |

**Table S7:** Cells in hydrogel reagents mix

| **3-D Life Dextran- PEG Hydrogel SG Kit (Cellendes) catalogue number: G92-1** | |
| --- | --- |
| **Reagents** | **Volumes for 100 µL gel (µL)** |
| Water | 35.3 |
| 10x CB, pH 7.2 | 8 |
| SG Dextran (30 mmol/L SH reactive groups) | 6.7 |
| Cell suspension | 40 |
| PEG-Link (20 mmol/L SH groups) | 10 |
| Total | 100 |

**Table S8:** PCR sample preparation:

Using Kit: Qiagen Taq PCR mix

All reagents are thawed on ice.

| **Reagent mix** | **Per reaction (uL**) |
| --- | --- |
| Taq PCR Mix. | 10 |
| Forward Primer (10 uM) | 1 |
| Reverse Primer (10 uM) | 1 |
| cDNA | 2 |
| NFW | 6 |
| Total | 20 |
